# Resolution of metastatic neck nodes associated with a periauricular cutaneous squamous cell carcinoma after intranodal injection of talimogene laherparepvec

**DOI:** 10.1016/j.jdcr.2024.02.008

**Published:** 2024-02-18

**Authors:** Yonglu Che, Curtis Chong, Marketa Limova, Laura Morris, Sunil Arani Reddy, Anne Lynn S. Chang

**Affiliations:** a Department of Dermatology, Stanford University School of Medicine, Redwood City, California; b Department of Medical Oncology, Stanford University School of Medicine, Redwood City, California; c Department of Dermatology, University of California San Francisco, Fresno, California; d Department of Medical Oncology, Stanford University School of Medicine, Stanford, California

**Keywords:** chronic lymphocytic leukemia, cutaneous oncology, cutaneous squamous cell carcinoma, herpes, immunotherapy, metastasis, squamous cell carcinoma, talimogene laherparepvec

## Introduction

Metastatic cutaneous squamous cell carcinomas (CSCCs) in patients with dysregulated immune systems, such as those with chronic lymphocytic leukemia (CLL), are a challenging yet growing group of patients, especially when they are not responsive to checkpoint immunotherapies. Talimogene laherparepvec (TVEC) is an oncolytic herpes virus approved by the US Food and Drug Administration in 2015 for melanoma recurrent after initial surgery and delivers human granulocyte-macrophage colony-stimulating factor leading to antitumor immune response.^1^ Although activity has been noted in nonmelanoma skin cancers, such as Merkel cell or mucosal head and neck squamous cell carcinomas, only one case report is currently in the literature of a CSCC response to TVEC with a duration of response of approximately 2 years.^2^

Here, we describe a case of a patient with CLL with recurrent and metastatic CSCC that progressed despite checkpoint inhibition, but experienced resolution of metastatic neck nodes after off-label TVEC therapy.

## Case report

A man in his 70s with CLL on ibrutinib presented to dermatology clinic with a 4-cm left periauricular ulcer, which had been biopsied and confirmed as CSCC.

**Fig 1:**
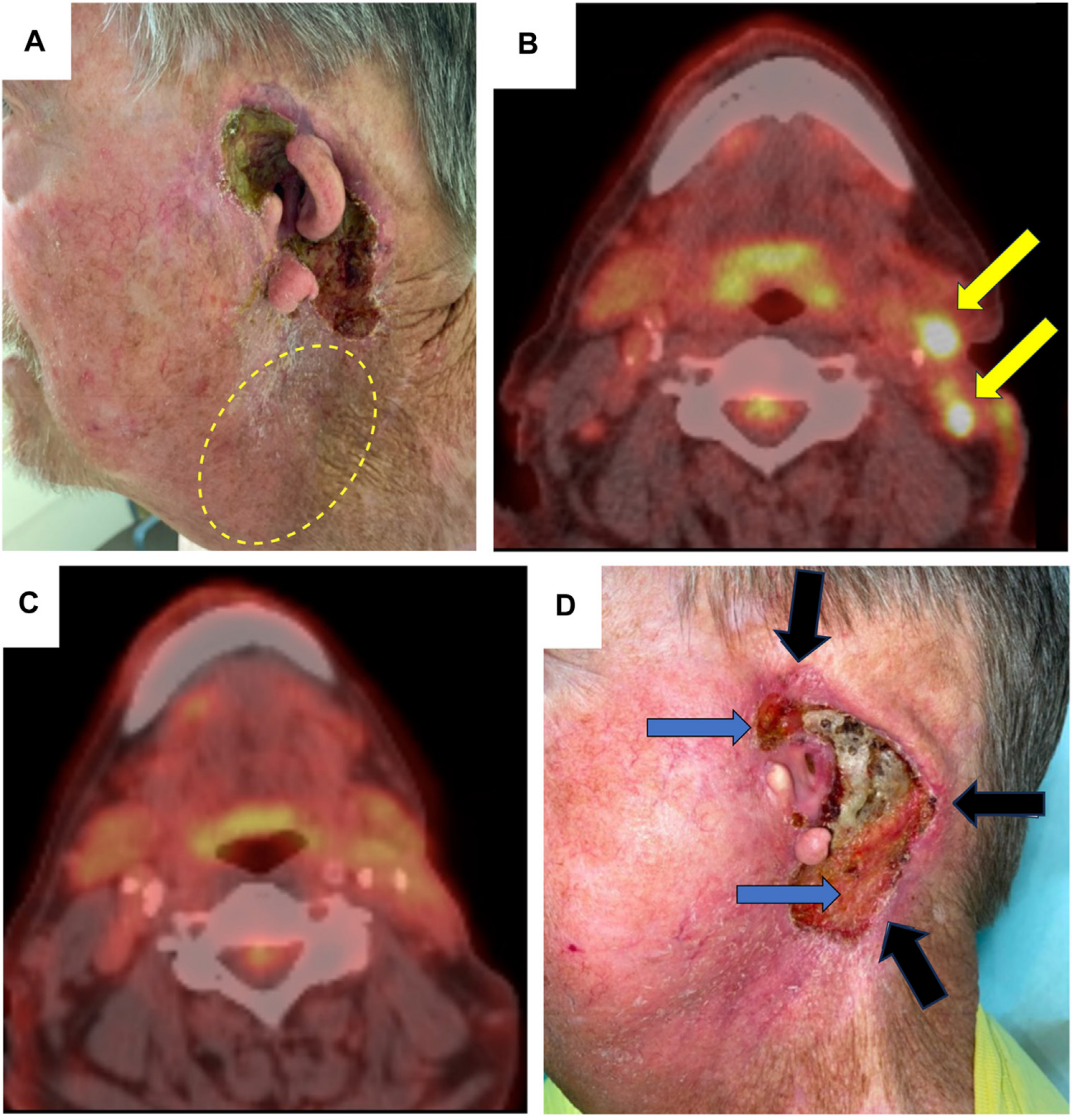
Metastatic cutaneous squamous cell carcinoma before and after treatment with talimogene laherparepvec (TVEC). **A**, Clinical photograph before treatment with TVEC. *Yellow dashed circle* indicates the area of palpable lymph nodes, and periauricular ulceration with erosion of cartilage is visible. **B**, *Yellow arrows* indicate pretreatment location of positron emission tomography (PET)-avid lymph nodes. **C**, PET scan about 6 months after last TVEC dose indicates continued clearance of the neck nodes (no avid neck lesions). **D**, Clinical photograph taken about 6 months after the last injection of TVEC. *Black arrows* indicate areas of scout punch biopsies which showed no CSCC. *Blue arrows* indicate areas of granulation tissue filling in areas of prior deeper defect without clinical recurrence.

The patient recalled that he had presented to an outside clinic several years ago for a new lesion on his left temple, which was biopsied and found to be CSCC. He underwent an excision, which subsequently required a left sided parotidectomy with left side of the neck lymph node dissection and adjuvant radiation. Eight months later, the patient reported a new nodule in front of his left ear, which was biopsied and confirmed as a recurrent CSCC, leading to a near-total left auriculectomy with positive margins, and subsequent treatment with the checkpoint inhibitor, pembrolizumab. After 4 months of pembrolizumab, the patient developed 2 palpable left cervical lymph nodes, with fine-needle aspirate confirming CSCC (Fig 1, *A* and *B*).

The patient consulted medical oncology and was offered systemic chemotherapy, but the patient declined because of concern for side effects. He was referred to clinical trials, but again declined. Off-label TVEC based on a case report for metastatic CSCC^2^ was discussed with the patient, including observed side effects, and he requested this option despite knowing that there are no systematic studies on response rate and safety in CSCC to date. The patient received 11 cycles of TVEC over 5 months, with first and second dose separated by 3 weeks and subsequent doses every 2 weeks. Dosing was 10^6^ plaque forming units (PFUs) × 2 mL per cycle for 4 cycles to ensure tolerability, followed by 10^8^ PFU × 3 mL per cycle for 5 cycles, then 10^8^ PFU × 4 mL per cycle for 2 cycles (maximum dose per cycle was 10^8^ PFU × 4 mL; total cumulative dose 23.08 mL × 10^8^ PFU). Injections were placed into the 2 neck nodes, although not the periauricular skin ulcer. We reasoned that treating the involved lymph nodes might theoretically lead to a stronger anticancer immune response. The neck nodes resolved on treatment and the periauricular skin ulcer started to granulate with decreased depth. The patient tolerated the treatment well, with low-grade fever after some injections (managed with acetaminophen), and an episode of cellulitis around the ear that resolved with oral tedizolid. The end point of treatment was the lack of specific findings for malignancy on positron emission tomography (PET) scan.

About 6 months after last TVEC injection, PET scan indicated complete resolution of the neck nodes (Fig 1, *C*). Three scouting 4 mm punch biopsies, performed to the superior, posterior, and inferior aspects of the remaining periauricular ulcer, returned negative for CSCC (Fig 1, *D*). In addition, there was 1 PET-avid nodule in the upper portion of the anterior aspect of the right lung suspicious for disease before TVEC (though not biopsied), which resolved after TVEC.

However, 9 months after the last TVEC treatment, subtle but persistent erosion on the inferior aspect of the periauricular ulcer prompted a punch biopsy, which revealed the presence of CSCC, although the neck nodes continued to be nonavid on PET scan. The patient restarted TVEC, with an additional 5 cycles every 2 weeks into the inferior aspect of the periauricular cutaneous ulcer (as the previously injected lymph nodes were no longer visible). TVEC reintroduction consisted of one full dose at 4 mL × 10^8^ PFU after which patient reported a mild headache. Hence, subsequent doses were reduced to 2 mL × 10^8^ PFU (total cumulative dose this second round of treatment was 12 mL × 10^8^ PFU). PET scan (day 471 after the first ever TVEC dose, and 10 days after last TVEC dose) indicated nonspecific changes on the periauricular skin and continued lack of avid lymph nodes. The patient opted to monitor for recurrence by clinical examination and imaging, and declined scouting biopsy of periauricular skin.

## Discussion

This case suggests that TVEC can have utility against metastatic CSCC in a patient with CLL, leading to a duration of response of approximately 6 months, and disease control for over 16 months. In comparison, a retrospective study of 10 patients on TVEC to treat melanoma of the head and neck reported median progression free survival of 10.8 months (95% CI, 2.2-19.4), although it is not clear if any of the patients had CLL.^3^ Additional cases of TVEC monotherapy in metastatic CSCC will better estimate the duration of response in immunocompromised individuals. We present this case as an example to support prospective studies of TVEC for patients with advanced CSCC who progress after checkpoint inhibitors, a growing area of unmet medical need.

Finally, we highlight the importance of close dermatologist collaboration with medical oncologists in cases where locally advanced CSCC is present (together with the metastatic disease), as detection of CSCC regrowth through careful clinical skin examination and early biopsy can identify persistence or recurrence, even before the lesion is detectable on imaging in some cases, so the patient can initiate retreatment without undue delay.

## Conflicts of interest

Dr Chang has served as a clinical investigator and consultant for Merck, Regeneron, Sun Pharma, Feldan, and Castle Biosciences. Dr Arani Reddy has served as a clinical investigator for BMS, Trisalus, and Delcath. Dr Chong has served as an advisory board member for AstraZeneca. The other authors have no conflicts of interest to declare.
